# Study on Decarburization Behavior in BOF Steelmaking Based on Multi-Zone Reaction Mechanism

**DOI:** 10.3390/ma18194599

**Published:** 2025-10-03

**Authors:** Zicheng Xin, Wenhui Lin, Jiangshan Zhang, Qing Liu

**Affiliations:** 1 State Key Laboratory of Advanced Metallurgy, University of Science and Technology Beijing, Beijing 100083, China; sklxzc@163.com (Z.X.); zjsustb@163.com (J.Z.); 2 School of Automation and Electrical Engineering, University of Science and Technology Beijing, Beijing 100083, China; 3 Jiangsu Jinheng Information Technology Co., Ltd., Nanjing 210031, China

**Keywords:** BOF steelmaking, multi-zone reaction mechanism, decarburization behavior, jet impact

## Abstract

In this study, the decarburization behavior in basic oxygen furnace (BOF) steelmaking was investigated based on the multi-zone reaction mechanism. The contributions of the main reaction zones to decarburization were clarified, and the effects of key factors—including the effective reaction amount in the main reaction zones, the post combustion ratio (PCR) in auxiliary reaction zones, and the carbon content of scrap steel—on decarburization behavior were quantitatively analyzed. The results indicate that decarburization predominantly occurs in the jet impact reaction zone (approximately 76% of the total decarburization), followed by the emulsion and metal droplet reaction zone (approximately 14%) and the bulk metal and slag reaction zone (approximately 10%). Variations in the effective reaction amount for the main reaction zones significantly affect both the decarburization rate and the endpoint carbon content, with the direct oxidation decarburization reaction in the jet impact reaction zone being the dominant factor. In addition, the PCR in the gas homogenization zone of the auxiliary reaction zones determines the distribution ratio of effective reaction oxygen, while the melting behavior of scrap steel in the metal homogenization zone plays a critical role in the precise control of the endpoint carbon content. This study provides a quantitative elucidation of the effects of different reaction zones on decarburization behavior, offering a foundation for the precise control of endpoint carbon content in BOF steelmaking.

## 1. Introduction

Basic oxygen furnace (BOF) steelmaking, with its high efficiency, low cost, and strong production adaptability, plays a dominant role in the steel industry, accounting for more than 70% of the global crude steel production [1,2,3,4]. Accurate prediction and control of the end-point carbon content in BOF steelmaking has consistently been an objective for precise steelmaking operations. The BOF smelting process involves gas-slag-metal multiphase coupled reactions as well as complex mass and heat transfer behaviors, and is influenced by fluctuations in input materials and differences in operation processes, which together pose challenges for the precise control of carbon content in molten steel. Therefore, a thorough analysis of the BOF decarburization process and a clear understanding of the effect of various factors on decarburization behavior are of great significance for enhancing process stability and reducing production costs.

Existing studies on the modeling of the BOF decarburization process can generally be categorized into two major types: (i) data-driven models based on statistical regression or machine learning algorithms [5], and (ii) mechanism models based on metallurgical reaction principles [6]. In terms of data-driven modeling, Gu et al. [7] developed an improved case-based reasoning model using time-series data to predict the end-point carbon content and temperature in converter. Zhang et al. [8] developed a cubic function model to predict the end-point carbon content in BOF steelmaking. Gao et al. [9] established a static control model for BOF steelmaking based on wavelet transform weighted twin support vector regression. The results show that within the prediction error range of ±0.005% for carbon content and ±10 °C for temperature, the hit ratios have reached 92% and 96%, respectively. Considering the limited predictive performance of traditional global models in handling multiple operating conditions, Peng et al. [5] proposed an endpoint prediction approach for BOF steelmaking based on just-in-time learning and local model integration, and the model accuracy was verified using actual data. Other researchers have also developed BOF endpoint prediction models based on algorithms such as eXtreme Gradient Boosting [10], robust relevance vector machines [11], and genetic algorithm-optimized kernel partial least squares regression [12].

In terms of mechanism modeling and dynamic control, Zhang et al. [13] established a converter smelting process model based on the coupling reaction mechanism. Liu et al. [14] proposed a new algorithm to control the endpoint carbon content of BOF based on off-gas analysis. Yue et al. [15] established the endpoint prediction model for BOF based on the sub-lance control, the decarburization index equation, the thermal equilibrium and the thermo-dynamical equation. Kadrolkar and Dogan [16] developed a mathematical model to predict the decarburization rate within individual droplets in the emulsion zone and investigated the contribution of decarburization rate in the emulsion zone to the overall decarburization rate using industrial data [17,18]. The results indicated that 5 to 75 pct of total decarburization takes place in the emulsion zone. Rout et al. [19] developed a decarburization dynamic model of basic oxygen steelmaking process based on multizone reaction kinetics. The results showed that approximately 76% of the decarburization occurred in the emulsion zone, while 24% occurred in the jet impact zone. Dogan et al. [20,21,22] divided the BOF into the bath zone and the emulsion zone, and developed a comprehensive model describing the decarburization reactions. The model was validated using data measured in a 200 t BOF [17,18], and the results indicated that approximately 45% of the decarburization occurred in the emulsion zone, while 55% occurred in the impact zone during the main blow. Dumont-Fillon et al. [23] and Meyer et al. [24] estimated the total decarburization in the molten bath based on the principle of carbon mass conservation by continuously integrating the decarburization rate during the BOF blowing process, thereby achieving continuous prediction of the carbon content evolution of the molten bath. Glasgow et al. [25] analyzed decarburization curves from a large number of industrial heats and proposed an exponential decay model for decarburization rate, which was used to predict the late-stage and endpoint carbon content in the BOF. Meanwhile, they introduced a single-point correction algorithm to adjust the predicted curves. Uemura [26] improved the single-point correction algorithm to a multi-point correction algorithm based on Glasgow’s research. Lin et al. [27] established an improved exponential model for carbon prediction in the end blowing stage of basic oxygen furnace converter based on “critical carbon content fitting + update curves simultaneously” algorithm. Xin et al. [28] developed a comprehensive model based on multi-zone reaction mechanism and verified this model using actual production data.

Data-driven models typically rely on large datasets and employ statistical analysis or machine learning algorithms for model establishment, without delving deeply into the metallurgical reaction mechanisms. Their advantage lies in achieving high predictive accuracy through advanced data analysis and machine learning techniques when sufficient data are available, which makes them promising for industrial applications. In contrast, the mechanism model is established based on the fundamental principles of steelmaking reactions. Among them, thermodynamic and kinetic parameters can be obtained through experimental measurement or theoretical derivation, and some parameters that are difficult to measure directly can also be determined by fitting industrial data. Such models are suitable for elucidating metallurgical reaction mechanisms, characterizing the evolution of process parameters, and performing both static calculations of process outcomes and dynamic simulations of process operations. Although significant progress has been made in modeling the BOF decarburization process and analyzing multi-reaction zone mechanisms in recent years, several limitations remain. In particular, the varying contributions of different reaction zones across different blowing stages have not been systematically evaluated. Moreover, existing studies have not conducted sensitivity analyses based on multi-zone reaction models, and the extent to which variations in key parameters of different reaction zones affect decarburization behavior has not been quantitatively assessed.

To address the above issues, the effects of the main reaction zones on the decarburization contribution and decarburization rate were investigated based on the multi-zone reaction mechanism comprehensive model developed in our previous study [28]. In addition, the effects of the effective reaction amount in the main reaction zone, the post combustion ratio (PCR) in the auxiliary reaction zone, and the carbon content of scrap in the auxiliary reaction zone on decarburization behavior were systematically examined, thereby providing a theoretical foundation for the precise control of carbon content in BOF steelmaking process.

## 2. Method and Model

**Figure 1:**
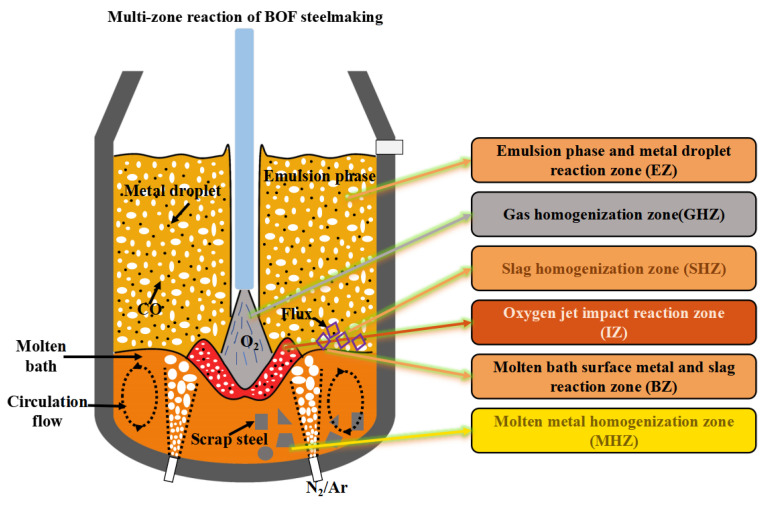
Schematic diagram of multi-zone reaction division in BOF steelmaking [28].

The top-blown oxygen jet serves as the primary driving force in BOF steelmaking reactions, with its impact zone being one of the most critical reaction zones during the blowing process. Simultaneously, the jet’s impact generates numerous molten metal droplets that splash into the slag, triggering slag–metal reactions to form an emulsified gas-slag-metal phase. The combined effects of top and bottom blowing create a circulating flow of molten metal, causing relative movement of slag-metal on the molten bath surface. This facilitates slag–metal reactions in the surface zone while promoting homogenization between slag phases and metallic phases. Additionally, a fraction of oxygen at the jet’s edge is utilized to initiate secondary combustion reactions in the furnace gas. Meanwhile, in our previous study [28], based on the metallurgical reaction mechanisms and process characteristics of the BOF steelmaking process, the multiphase reaction zones inside the BOF were divided into six zones: the jet impact reaction zone (IZ), the bulk metal and slag reaction zone (BZ), the emulsion and metal droplet reaction zone (EZ), the gas homogenization zone (GHZ), the metal homogenization zone (MHZ), and the slag homogenization zone (SHZ), as shown in Figure 1. Among these zones, IZ, BZ, and EZ are the main reaction zones, while GHZ, MHZ, and SHZ serve as auxiliary reaction zones. Based on this, combined with the metallurgical thermodynamic and kinetic principles, the reactions in all six zones were systematically analyzed, and a multi-zone reaction mechanism comprehensive model was developed. For each reaction zone, both thermodynamic and kinetic principles were systematically considered. From a thermodynamic perspective, the Gibbs free energy minimization method, partition Gibbs energy approach, and FactSage 7.0 thermodynamic software were employed to calculate the equilibrium state of oxidation and reduction reactions. From a kinetic perspective, reaction rates were modeled through effective mass transfer coefficients, diffusion coefficients, and circulation renewal rates, considering the coupling effects of bottom and top blowing. The analysis of each reaction zone and the development of the comprehensive model have been detailed in Ref. [28] and will not be repeated here.

Based on the multi-zone reaction mechanism comprehensive model and its calculation results, the effect of the main reaction zone on the decarburization contribution and decarburization rate was assessed considering four typical industrial heats (denoted as Heat A, Heat B, Heat C, and Heat D), covering three representative steel grades: low-carbon, medium-carbon, and high-carbon steels (detailed information of these heats is provided in Ref. [28] and Section 3). Subsequently, taking Heat B under normal blowing conditions as a case study, the effects of the effective reaction amounts in the main reaction zones, the PCR in the auxiliary reaction zones, and the carbon content of the scrap steel in the auxiliary reaction zones on the decarburization behavior were further investigated.

## 3. Results and Discussion

### 3.1. Effect of Main Reaction Zone on Decarburization Process

**Figure 2:**
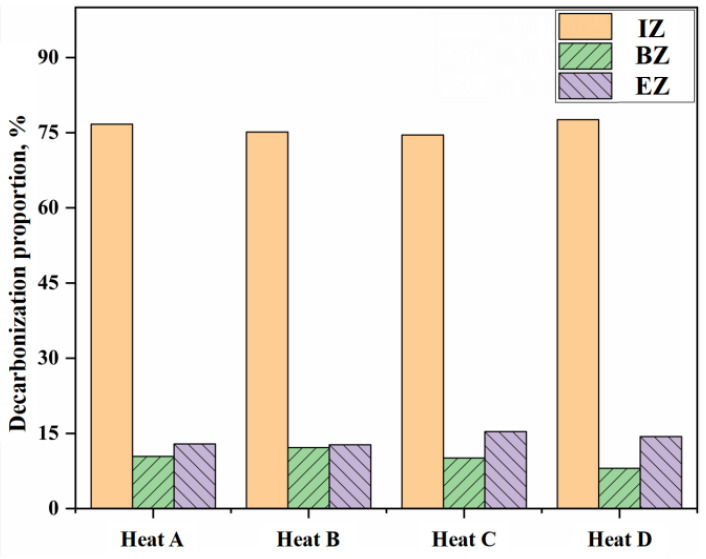
Decarburization proportion of different reaction zones in BOF.

In our previous study [28], the overall calculation deviation of the carbon content in the BOF steelmaking process could be controlled at a relatively good level by the multi-zone reaction mechanism comprehensive model. Based on this, the effects of different reaction zones on decarburization behavior were investigated. From a multi-zone reaction mechanism perspective, the decarburization reaction in the BOF primarily occurs in three forms: the gas–liquid reactions in the IZ, the slag-metal reactions in the BZ, and the metal droplet reactions in the EZ. Accordingly, the decarburization can be partitioned into three corresponding parts based on the zones in which they occur. Simulation results from the multi-zone reaction mechanism comprehensive model indicate the proportion of decarburization contributed by each of these three zones, as illustrated in Figure 2.

**Figure 3:**
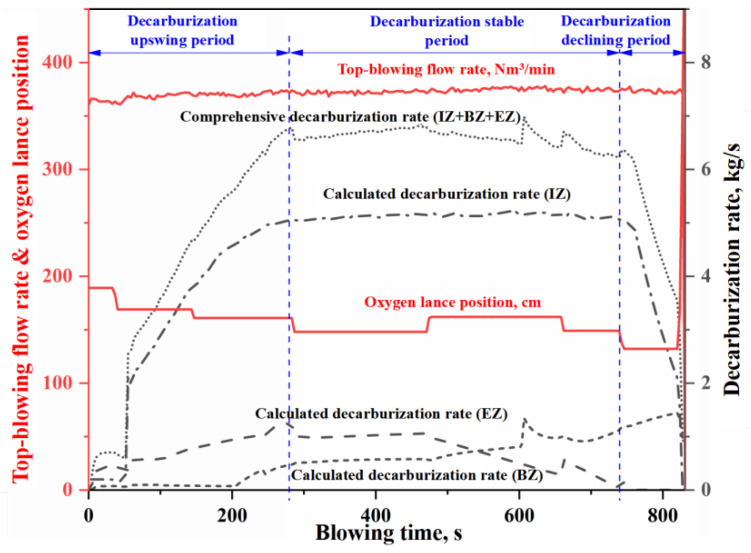
Decarburization rate curve in each zone (Heat A).

**Figure 4:**
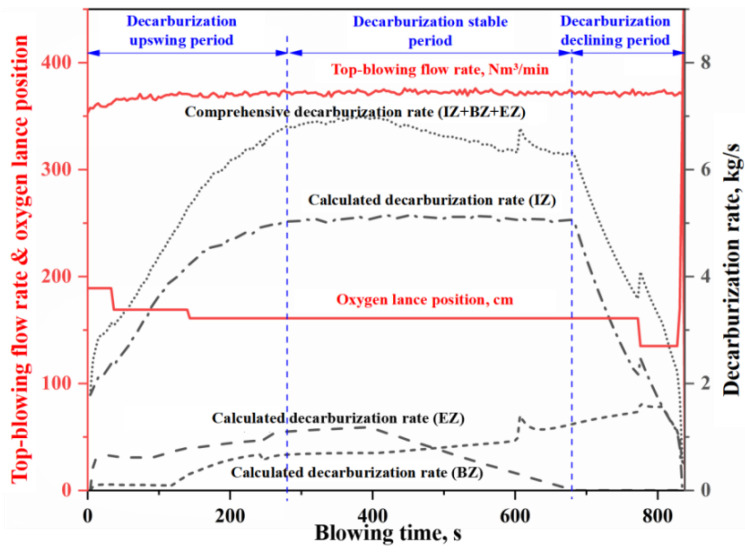
Decarburization rate curve in each zone (Heat B).

**Figure 5:**
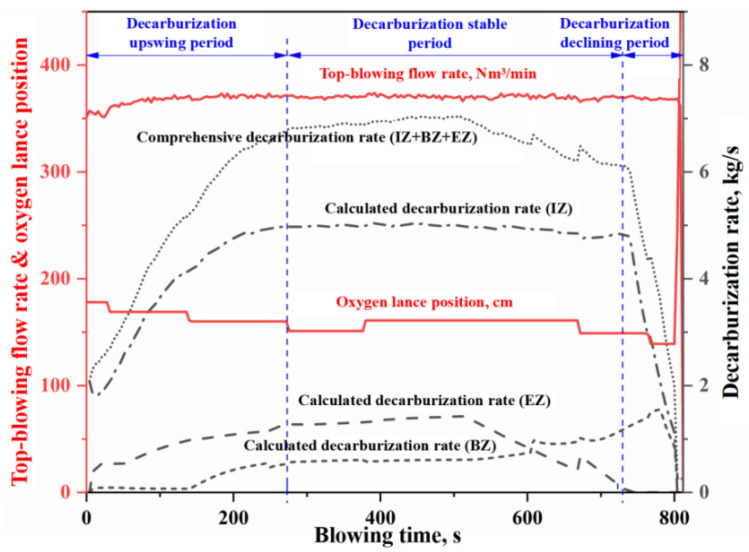
Decarburization rate curve in each zone (Heat C).

**Figure 6:**
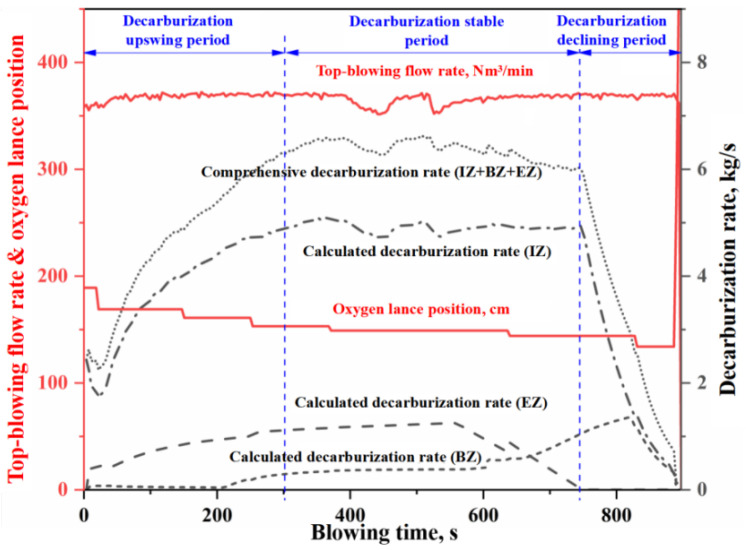
Decarburization rate curve in each zone (Heat D).

In this study, as shown in Figure 2, the average contributions of the IZ, the BZ, and the EZ to total decarburization across four heats are 76%, 10%, and 14%, respectively. It can be seen that the relative contributions of these zones to BOF decarburization decrease in the order IZ > EZ > BZ. Figure 3, Figure 4, Figure 5 and Figure 6 present the continuous variation in decarburization rates in each reaction zone for Heat A, Heat B, Heat C, and Heat D, along with process parameters such as top-blowing flow rate and oxygen lance position.

As shown in Figure 3, Figure 4, Figure 5 and Figure 6, due to differences in the materials and process operating conditions, the transient profiles of the decarburization rate curves for heats A, B, C, and D vary. However, many common features can be observed in the overall contour of the decarburization rate curves. Specifically: 

(i) the gas–liquid reaction in the IZ dominates decarburization in the BOF, and the profile of the decarburization rate curve in the IZ largely determines the overall shape of the comprehensive decarburization rate curve. Based on the profile of the comprehensive decarburization rate curve (or that of the IZ decarburization rate curve), the decarburization process can be divided into three stages: decarburization upswing period, decarburization stable period, and decarburization declining period, corresponding to the conventional early stage, middle stage, and late stage of blowing.

(ii) The decarburization rate curve in the IZ exhibits three distinct stages, characterized by a gradual rise, a steady plateau, and a rapid decline. During the upswing and stable periods, the decarburization reaction is controlled by the mass transfer of oxygen, and the molten steel in the IZ is partially decarbonized. However, during the declining period, the decarburization reaction is controlled by the mass transfer of carbon, and the molten steel in the IZ becomes fully decarburized. The gradual rise in the curve during the early stage is mainly attributed to the competitive oxidation among silicon, manganese, and carbon, where a portion of the effective reaction oxygen is consumed in oxidizing silicon and manganese. As the content of silicon and manganese decrease, the decarburization rate gradually increases until desiliconization is complete and enters the steady decarburization stage. During the stable period, the decarburization rate in the IZ is completely controlled by the oxygen supply flow rate and is essentially unaffected by other factors. Abnormal fluctuation of oxygen supply flow will lead to fluctuation of decarburization rate (e.g., two abnormal fluctuations around 450 s and 550 s in Figure 6). When the carbon content decreases below the critical threshold for the carbon-oxygen mass transfer transition in gas–liquid reactions, decarburization becomes controlled by carbon mass transfer. The decarburization rate declines rapidly with decreasing carbon content, exhibiting distinct decline trends depending on oxygen lance position adjustments (as evidenced by the significant adjustment of oxygen lance position around 770 s in Figure 4, which caused a significant mutation in the decarburization decline curve).

(iii) The decarburization rate curve of the EZ exhibits an upward trend during the upswing period and the first half of the stable period, followed by a rapid decline in the latter half of the steady stage, while no decarburization occurs in the EZ during the declining period. In the upswing period and the first half of the stable period, the carbon content of the metal droplets is high, and the decarburization reaction is controlled by oxygen mass transfer in the slag. The total decarburization in the emulsion phase increases with the slag amount and the accumulation of FeO in the slag. In the latter half of the stable period, as the carbon content in the metal droplets decreases, the decarburization reaction is controlled by carbon mass transfer. At this time, the decarburization rate in the EZ decreases rapidly with the decrease in carbon content in the metal droplets. Since the metal droplets in the emulsion phase are primarily generated by jet impact, and the molten steel in the IZ is already fully decarburized during the declining period, the metal droplets entering the slag at this stage contain almost no carbon. Therefore, decarburization does not occur in the EZ during the declining period.

(iv) The decarburization rate curve of BZ shows a three-stage trend of slow rise, rapid rise and rapid decline. In the early stage of blowing, the decarburization reaction on the surface of the molten bath is inhibited by the competition between silicon and carbon oxidation in slag-metal reaction on the surface of the molten bath, and the decarburization rate is very small. With the removal of silicon, the decarburization rate of the slag-metal reactions increases; however, due to the mass transfer limitation of FeO in the slag, the increase in decarburization rate is slow. When the decarburization rate in the EZ begins to decline rapidly as the carbon content of the metal droplets decreases (reaching the critical value for the transition of carbon-oxygen mass transfer in the slag-metal reactions), the FeO consumption of decarburization reaction in the EZ decreases, which means that the FeO amount in the slag increases rapidly. At this time, the carbon content of molten metal in the molten bath is higher than that of the metal droplet (it does not reach the critical value for the transition of carbon-oxygen mass transfer in the slag-metal reactions). Meanwhile, after 600 s, the strong stirring induced by bottom blowing increases the renewal rate of the slag-metal circulation at the molten bath surface. The increase in FeO content in slag and slag–metal circulation velocity lead to a rapid increase in decarburization rate at the molten bath surface. When the carbon content of the molten metal in the molten bath decreases below the critical value for the transition of carbon-oxygen mass transfer in the slag-metal reaction, the decarburization reaction becomes controlled by carbon mass transfer, and the decarburization rate of the slag-metal reaction decreases rapidly with the decrease of carbon content. For the three stages of the BOF decarburization process, the curve morphology during the overall decarburization upswing period is jointly determined by the decarburization in IZ, EZ, and BZ. The curve morphology during the stable period is mainly influenced by the decarburization in EZ and BZ, whereas the declining period is determined by the decarburization in IZ and BZ.

**Figure 7:**
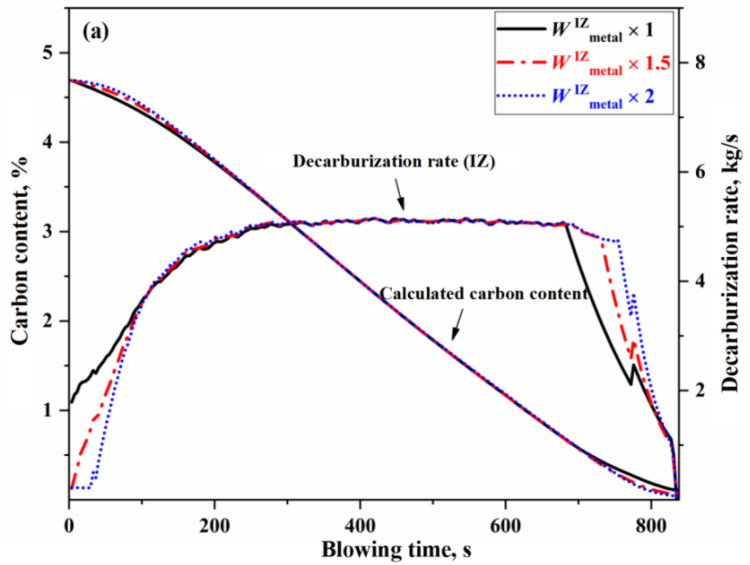

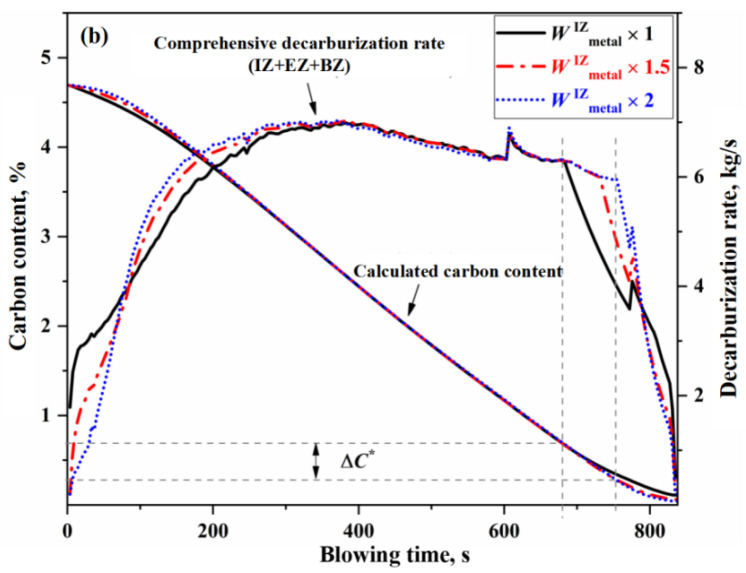
Effect of effective reaction amount in IZ on decarburization rate and carbon content control. (**a**) Decarburization rate (IZ), and (**b**) Comprehensive decarburization rate rate (IZ+EZ+BZ).

**Figure 8:**
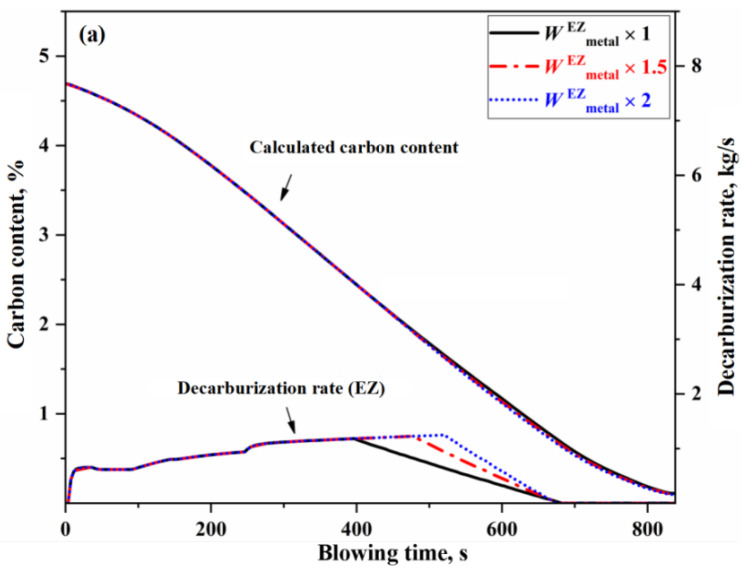

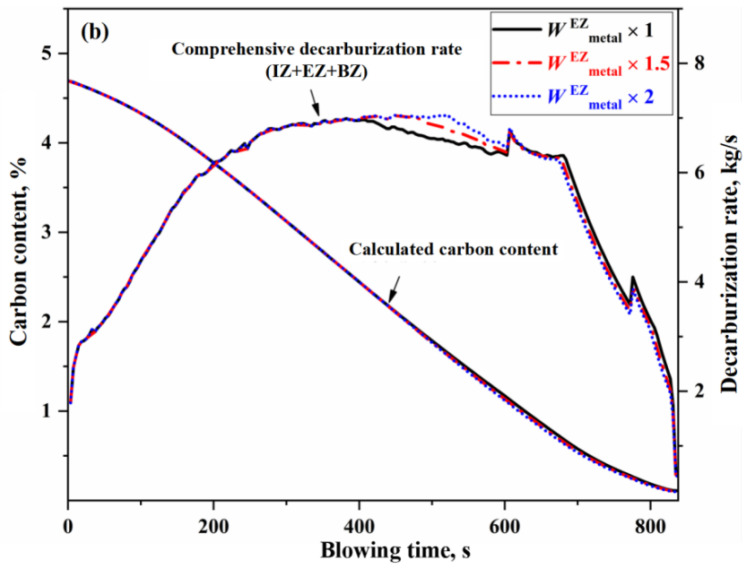
Effect of effective reaction amount in EZ on decarburization rate and carbon content control. (**a**) Decarburization rate (EZ), and (**b**) Comprehensive decarburization rate (IZ+EZ+BZ).

**Figure 9:**
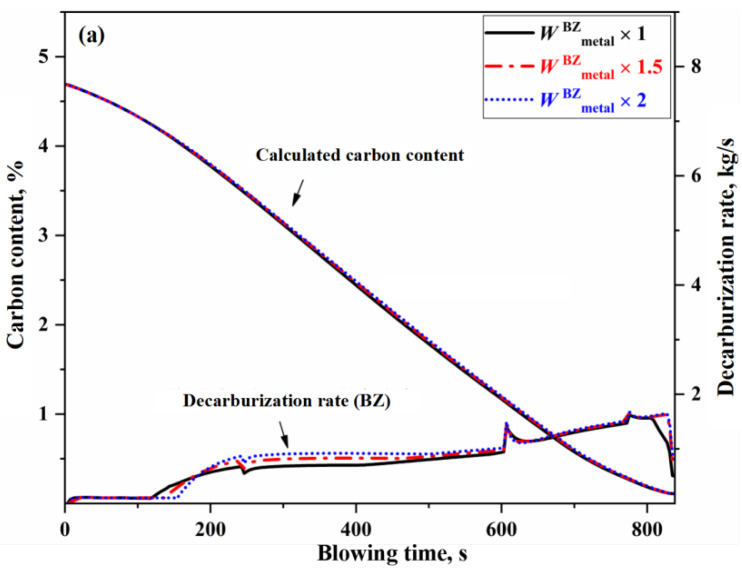

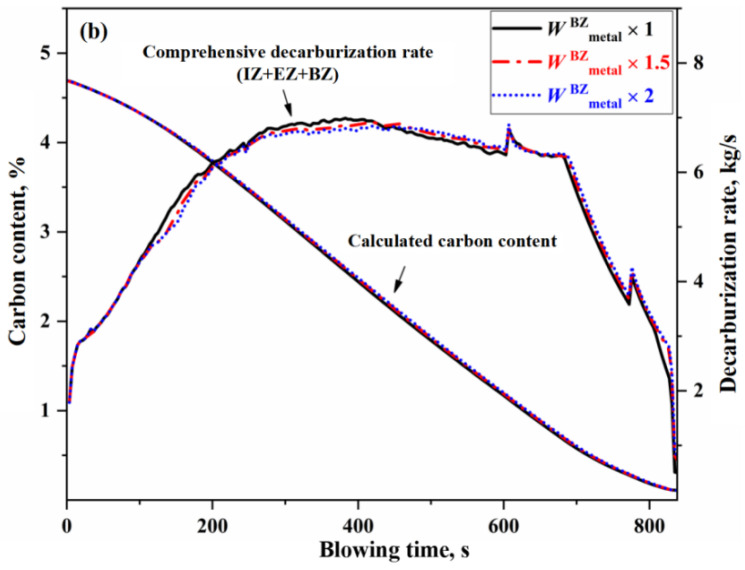
Effect of effective reaction amount in BZ on decarburization rate and carbon content control. (**a**) decarburization rate (BZ), and (**b**) Comprehensive decarburization rate (IZ+EZ+BZ).

From a kinetic perspective, the decisive factors influencing the decarburization reaction rate are the effective reaction amounts in the IZ, EZ, and BZ. The change of effective reaction amount in each zone will have different effects on the decarburization curve. Taking normal blowing Heat B as an example, this study changes the effective reaction amount of one zone (that is, the effective reaction amount of the zone is 1 time, 1.5 times and 2 times of the original effective reaction amount under the condition that other conditions remain unchanged), and the results are shown in Figure 7, Figure 8 and Figure 9. Since the decarburization amount in the IZ accounts for the largest proportion, the change of effective reaction amount in the IZ has the most significant effect on the decarburization rate curve and carbon content control, which can be reflected in the comparison of deviation degree of each curve from Figure 7, Figure 8 and Figure 9.

In Figure 7, the change of effective reaction amount in the IZ causes a significant fluctuation in the upswing and declining periods of the decarburization rate curve, while having almost no effect on the decarburization rate curve in the stable period. As a result, fluctuations in carbon content during the early and late stages of blowing reach −0.095 to 0.10%. When the effective reaction amount in the IZ is increased by one time, the endpoint carbon content decreases by 0.066%, and the corresponding critical carbon content at which the decarburization rate begins to decline also changes markedly. As shown in Figure 7, the variation in the critical carbon content was 0.41% ($\Delta C^{*}\approx 0.41\;\%$), which is closely related to the kinetic conditions. This indicates that reactions in the IZ play a crucial role in determining the critical carbon content entering the decarburization declining period. Furthermore, as the effective reaction amount in the IZ increases, the variation gradients of the decarburization rate curve during the upswing and declining periods also increase. That is, the larger the effective reaction amount in the IZ, the steeper the decarburization rate curve in the early and late stages of blowing.

In Figure 8, the changes of effective reaction amount in the EZ (i.e., the amount of metal droplets) primarily affect decarburization during the latter half of the stable period. Specifically, after the decarburization of the droplets becomes controlled by carbon mass transfer, the changes of effective reaction amount in the EZ lead to significant variations in the decarburization rate curve. Meanwhile, due to its effect on the carbon content, the later portions of both the carbon content curve and the decarburization rate curve (corresponding to the declining period) exhibit a slight overall shift. When the effective reaction amount in the EZ is increased by one time, the carbon content curve shifts by −0.051% to 0, with the endpoint carbon content decreasing by 0.013%. During the upswing period and the first half of the stable period of decarburization, the decarburization rate in the EZ is primarily controlled by oxygen mass transfer in the slag, so variations in the amount of metal droplets have little effect on decarburization. In the declining period, the molten metal in the IZ has already been fully decarburized through the gas–liquid reaction; therefore, the metal droplets in the emulsion phase no longer undergo decarburization during this stage.

Unlike the EZ, where one of the reactants (metal droplets) is a discrete phase while the slag is a gas-slag continuous phase, both reactants in the BZ, namely the molten metal and slag, are continuous phases. As a result, variations in the effective reaction amount in this zone influence decarburization throughout the entire BOF steelmaking process, as shown in Figure 9. Variations in the effective reaction amount in the BZ cause an overall shift in both the decarburization rate curve and the carbon content curve throughout the steelmaking process. When the effective reaction amount in the BZ is increased by one time, the carbon content curve shifts by −0.002% to 0.046%, with the endpoint carbon content decreasing by 0.011%. In summary, the primary factor affecting carbon content control in BOF steelmaking is the direct oxidation decarburization reaction in the IZ, followed by the decarburization reaction of metal droplets in the EZ. The slag-metal reaction in the BZ has the least impact on carbon content control.

For the IZ, decarburization accounts for the largest proportion of the total decarburization (approximately 76%). Variations in the kinetic conditions of this zone have the greatest impact on both the decarburization rate and carbon content control during the decarburization upswing and declining periods. Based on analyses of the top-blowing jet impact behavior and the kinetics of the IZ [28,29], from BOF operation perspective, the primary method to control the effective reaction amount in the IZ (i.e., the weight of molten steel displaced by the impact crater) is by regulating the top-blowing flow rate and oxygen lance position, as well as maintaining an appropriate slag amount (since slag thickness significantly affects the impact depth). This enables the formation of a suitably sized impact crater and an appropriate weight of molten steel displaced by the jet impact.

For the EZ, its influence on the decarburization rate and carbon content control primarily occurs during the latter half of the stable period up to the onset of the declining period. During this period, decarburization in the EZ is controlled by carbon mass transfer, and the amount of metal droplets has a significant impact on both the decarburization rate and the carbon content. The amount of metal droplets generation is determined by the blowing number and the oxygen flow rate, with the blowing number being associated with the dimensionless lance position [28,30]. Therefore, the critical factor in controlling the effect of the EZ on decarburization is the regulation of lance position and oxygen flow rate during the latter half of the decarburization stable period. For the BZ, the reaction kinetics is mainly determined by bottom blowing [28], and less affected by top blowing. Consequently, the critical factor in controlling the effect of the BZ on decarburization is the regulation of bottom blowing.

### 3.2. Effect of Auxiliary Reaction Zone on Decarburization Process

**Figure 10:**
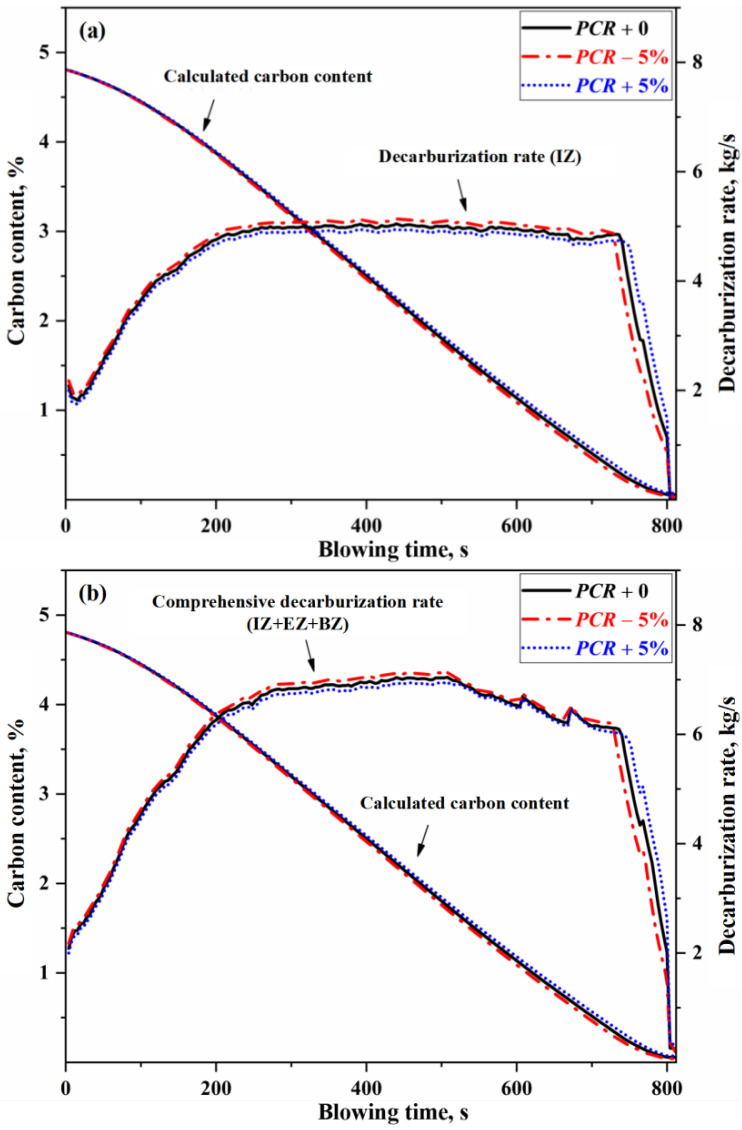
Effect of PCR on decarburization rate and carbon content control. (**a**) Decarburization rate (IZ), and (**b**) Comprehensive decarburization rate (IZ+EZ+BZ).

In the auxiliary reaction zone, secondary combustion reaction occurs in the GHZ. Different PCR methods correspond to different effective oxygen fractions available for reactions in the IZ. Under conventional operating conditions, the average PCR in top-blown oxygen converters fluctuates within approximately ±5%. When the total oxygen flow rate remains constant, an increase in the PCR implies a decrease in the effective oxygen fraction available for reactions in the IZ, whereas a lower PCR corresponds to a higher effective oxygen fraction. Similarly, taking Heat B as an example, the effect of different PCR on decarburization rate and carbon content control of converter under the condition of other unchanged conditions is shown in Figure 10.

In Figure 10, when the PCR fluctuates by ±5%, the decarburization rate in the IZ and the overall decarburization rate fluctuate by approximately ±0.09 kg/s, while the carbon content curve shifts by about ±0.04%. Regarding the final decarburization outcome, when the PCR increased by 5%, the endpoint carbon content increased by 0.016%, and when the PCR decreased by 5%, the endpoint carbon content decreased by 0.013%. Although the secondary combustion of converter off-gas itself does not directly participate in decarburization reactions, it determines the proportion of effective reaction oxygen in the IZ relative to total oxygen supply. Therefore, the PCR exerts a significant influence on the control of carbon content. The oxygen lance position is the primary factor influencing the post-combustion ratio [28]. Therefore, the key to controlling the PCR is to control the oxygen lance position.

In addition to the factors mentioned above, the effect of scrap steel melting reaction in the MHZ on carbon content control in the converter should also be considered. The effect of scrap steel on the carbon content of the molten bath can be attributed to two main aspects. Firstly, the wide variety of scrap steel types and the lack of routine composition measurements often result in significant deviations in the calculated total carbon mass (initial carbon content). This represents one of the primary reasons why most steelmaking plants cannot directly apply mechanism models to accurately control carbon content throughout the blowing process. This issue can mainly be mitigated through improved scrap steel classification and management. Secondly, if the size of the scrap steel is too large or the bottom-stirring process is not reasonable, some scrap steel is not fully melted at the end of the blowing process, which may adversely affect the control of endpoint carbon content in the converter. At the end of the blowing process, if a certain amount of scrap steel remains unmelted in the molten bath, it will continue to melt under thermal driving mechanisms after stopping blowing. The carbon from the scrap steel dissolving into the molten metal will increase its carbon content, thereby affecting the accurate control of both the endpoint temperature and carbon content of the molten steel.

**Figure 11:**
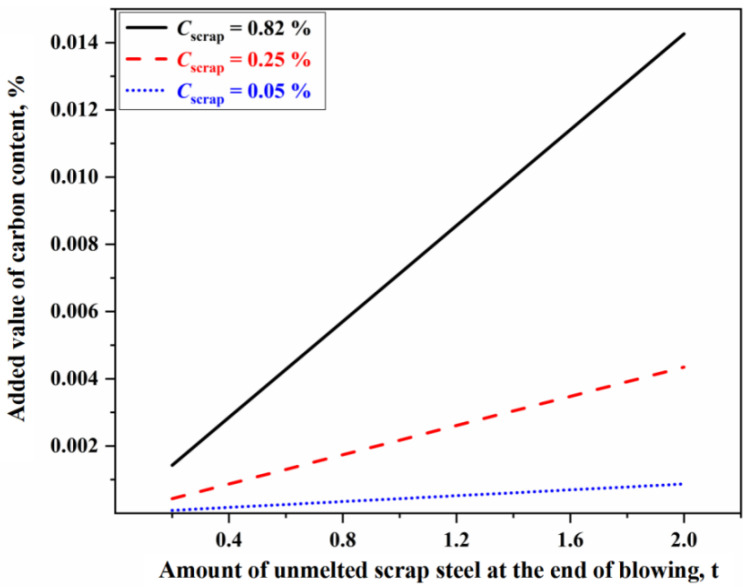
Effect of amount of unmelted scrap steel at the end of blowing on carbon content control.

In this study, the large-sized scrap steel primarily consisted of remaining sizable pieces from tundish and thick plate scrap steel, including hot-rolled low-carbon steel, carbon structural steel, and high-carbon hard wire steel. The average carbon content of the low-carbon, ordinary carbon, and high-carbon scrap steel was assumed to be 0.05%, 0.25%, and 0.82%, respectively. Based on these values, the relationship between the amount of unmelted scrap steel at the end of blowing process and the added value of final carbon content in the converter was determined, as shown in Figure 11. In Figure 11, the effect of different carbon content of scrap steel on the added value of carbon content in the molten bath is different. The higher the carbon content of scrap steel, the greater the influence. When high-carbon scrap steel ($C_{\mathrm{scrap}}=0.82\;\%$) is used, if the amount of unmelted scrap steel at the end of blowing exceeds 1.4 t, the carbon content in the molten bath increases by more than 0.01%, thereby exerting a non-negligible influence on the control of carbon content in the converter.

## 4. Conclusions

Based on the multi-zone reaction mechanism comprehensive model, this study investigated the effects of reactions in different zones on decarburization behavior and clarified the key factors influencing decarburization. The main research conclusions are as follows:

(1) The decarburization reaction mainly occurs in IZ, BZ, and EZ. Their contributions to overall decarburization decrease in the order IZ > EZ > BZ, with each zone accounting for 76%, 14%, and 10% of the total decarburization, respectively.
(2) The morphology of the decarburization rate curve in the IZ determines the overall profile of the comprehensive decarburization rate curve. Under the same oxygen blowing conditions, the larger the effective reaction amount in the IZ, the steeper the decarburization rate curve in the early and late stages of blowing, while having almost no effect on the decarburization rate curve in the stable period. When the effective reaction amount in the IZ is increased by one time, the endpoint carbon content decreases by 0.066%.
(3) The changes of effective reaction amount in the EZ (i.e., the amount of metal droplets) primarily affect decarburization during the latter half of the stable period, while has no effect on the upswing period and the first half of the stable period. The metal droplets in the emulsion phase no longer undergo decarburization during the declining period. When the effective reaction amount in the EZ is increased by one time, the endpoint carbon content decreases by 0.013%.
(4) The variation in the effective reaction amount in the BZ affects decarburization throughout almost the entire blowing process, but the overall impact is relatively small. When the effective reaction amount in the BZ is increased by one time, the endpoint carbon content decreases by 0.011%.
(5) When the PCR in the GHZ fluctuates by ±5%, the decarburization rate in the IZ and the overall decarburization rate vary by approximately ±0.09 kg/s, while the endpoint carbon content fluctuates between −0.013% and 0.016%. When the amount of unmelted scrap steel (high carbon scrap steel) in the MHZ at the end of blowing exceeds 1.4 t, the carbon content in the molten bath increases by more than 0.01%.

## Data Availability

The data presented in this study are available on request from the corresponding author due to privacy.
